# Process evaluation of an academic-community-government partnership to reduce liver diseases attributable to hepatitis B virus

**DOI:** 10.1186/s12913-022-08062-7

**Published:** 2022-05-26

**Authors:** Daisy Le, Min Jeong Jeon, Annie Coriolan Ciceron, Y. Tony Yang, Jane Pan, Hee-Soon Juon, Sherrie F. Wallington

**Affiliations:** 1 The George Washington University, School of Nursing, 1919 Pennsylvania Ave NW, Suite 500, Washington, DC 20006 USA; 2 The George Washington University, Milken Institute School of Public Health, 950 New Hampshire Ave NW #2, Washington, DC 20052 USA; 3 Hepatitis B Initiative of Washington DC (HBI-DC), 1725 I St NW #300, Washington, DC 20006 USA; 4 Department of Medical Oncology, Thomas Jefferson University, 834 Chestnut Street, Benjamin Franklin House, Suite 320, Philadelphia, PA 19107 USA

**Keywords:** Community-based participatory research, Community health partnerships, Community health research, Health disparities, Health outcomes

## Abstract

**Background:**

Racial/ethnic minorities have higher incidence and mortality rates of liver cancer, or hepatocellular carcinoma, than non-Hispanic Whites. As such, the Washington-Baltimore Metropolitan Area Hepatitis B Virus (WB-HBV) Demonstration Project, a community-based participatory research (CBPR)-driven academic-community-government (ACG) partnership, was established in 2019 to address disparities and implement strategies to improve the HBV screening and vaccination infrastructure for at-risk communities. CBPR is a partnership of community members, organizational leaders, and academic researchers with a common aim to collectively share and contribute their input at every phase of the project.

Herein, we describe the process evaluation of the WB-HBV Project and extract themes and insights to benefit future ACG partnerships and community-engaged research. The process evaluation has been conducted to determine whether CBPR-driven partnership and programmatic activities have been implemented as intended and have resulted in building expanded research capacity for future ACG partnership HBV community-level initiatives.

**Methods:**

A WB-HBV Project Task Force was convened and comprised of eight organizations: four community organizations, three government organizations, and one academic institution. Through a mixed-methods process evaluation, an online survey and key informant interviews were conducted to provide context for program implementation barriers and facilitators. Descriptive statistics were conducted, and interviews were recorded, transcribed, and thematically coded.

**Results:**

The survey was completed by 14 of 20 partnership members (70.0%): two academic, eight community, and four government members. Partnership members showed general agreement across 14 domains: organization and structure of meetings; trust; decisions; impact; general satisfaction; strategic planning; ACG policy impact; community-based participatory research and government; participation in meetings; assessment of participation; partnership operations and capacity; communication; challenges/limitations associated with ACG involvement; and benefits compared to challenges associated with ACG involvement. Qualitative interviews were conducted with 15 of the 20 members (75.0%): two academic, nine community, and four government members. Four themes emerged: partnership involvement, project goals and accomplishments, project challenges and barriers, and partnership involvement in government or policy.

**Conclusions:**

The process evaluation presents insights into developing strategies to enhance partnership functioning and increase the ability of present and future ACG partnerships to improve community health outcomes.

## Background

Community-based participatory research (CBPR) is a partnership of community members, organizational leaders, and academic researchers with a common aim to collectively share and contribute their knowledge and expertise to improve the health of community members [1]. Community members’ active and continued engagement in CBPR is crucial, especially because of challenges conducting research in underserved communities [2]. CBPR fosters equitable participation of each stakeholder from the academic, community, and government settings to enable collaborative changes that will improve the health of community members [3]; CBPR has been widely used in research and has been shown as an effective method to reduce cancer disparities [4]. However, challenges in building, engaging, and sustaining CBPR partnerships often exist [4]. Overcoming these challenges requires assessing program implementation barriers and facilitators among the collaborative partnership members working to improve community health.

Liver cancer, or hepatocellular carcinoma (HCC), is a leading cause of death worldwide, and approximately 24,000 men and 10,000 women are diagnosed with HCC each year in the US [5–7]. According to the Centers for Disease and Prevention (CDC), approximately 18,600 men and 9000 women die from HCC each year [7]. The risk for HCC increases with chronic hepatitis B virus (HBV) or hepatitis C virus (HCV) infection. Despite vaccination and treatment options, respectively, for HBV and HCV infections, racial/ethnic minorities have higher incidence and mortality rates than non-Hispanic Whites [5]. In the Washington-Baltimore Metropolitan Area (WBMA) from 2009 to 2015, the prevalence rates of HBV and HCV, respectively, were 6.1 and 3.8% among Asian-born immigrants and 3.7 and 2.8% for African-born immigrants [6]. Since most infected individuals show little to no symptoms until their liver disease is well advanced, they are often diagnosed with late-stage cancer that results in low survival rates and high mortality rates [5–7]. Due to the asymptomatic nature of HCC, it is crucial to screen and prevent complications from HCC among foreign-born individuals who have migrated from countries where hepatitis viruses are endemic.

The Washington-Baltimore Metropolitan Area Hepatitis B Virus (WB-HBV) Demonstration Project is an integrated, multijurisdictional community coalition that aims to deliver prompt, responsive, and efficient care for immigrants from countries where HBV infection is endemic (e.g., Asia and Africa). The overall objective of this collaborative academic-community-government (ACG) partnership is to implement strategies and interventions to improve HBV screening and vaccination infrastructure for at-risk communities in Washington, District of Columbia (DC); Maryland; and Virginia. It covers a cross-state area populated by residents identified by the CDC as being most at-risk for and affected by this disease. The WB-HBV Project Task Force comprises the National Institute of Diabetes and Digestive and Kidney Diseases within the National Institutes of Health, four community organizations (inclusive of primary care physicians, a virologist, and an infectious disease specialist), three local health departments, and the George Washington University research team. To assess program implementation barriers and facilitators among the collaborative partnership members, an external evaluation team was convened to develop and execute a process evaluation plan. The purpose of this article is to (a) describe the process evaluation of an ACG partnership created to address HBV health disparities in the WBMA, and (b) extract themes and insights to benefit future ACG partnerships and the field of CBPR.

## Methods

A WB-HBV Project Task Force was convened and comprised of eight local organizations: four community organizations, three government organizations, and one academic institution. Through purposeful sampling, these organizations were selected based on their historical and current efforts in providing HBV outreach, education, screening or testing, and linkage to care and treatment services to at-risk communities, particularly among the foreign-born population, in the WBMA. This cross-jurisdictional collaborative partnership was intentionally organized to develop a sustainable model to allow for HBV health information exchange between providers in the WBMA.

To conduct process evaluation, an external evaluation team approached task force members using an online survey and key informant interviews. This mixed-methods approach using closed and open-ended questions was implemented to quantitatively and qualitatively provide context for program implementation barriers and facilitators among members of the WB-HBV Project Task Force. The external evaluation team consisted of 3 individuals who were not directly involved in the implementation of the WB-HBV Project.

### Data collection and measures

#### Brief partnership interview survey

The quantitative evaluation survey was adapted from Israel and colleagues [8]. The original survey consisted of 110 items that assessed 15 domains. Items assessed included general satisfaction, trust, operations and capacity, and organization and structure of meetings [8]. To reduce participant burden, the external evaluation team identified and adopted 69 items from the original survey. A confidential online instrument assessed the organization and structure of meetings, trust, decisions, impact, general satisfaction, strategic planning, ACG policy impact, CBPR and government, participation in meetings, assessment of participation, partnership operations and capacity, communication, benefits of ACG involvement, challenges/limitations associated with ACG involvement, and benefits compared to challenges associated with ACG involvement. The majority of the responses were on a 5-point Likert scale: (1) strongly agree, (2) agree, (3) neutral, (4) disagree, and (5) strongly disagree. Some responses were on a 4-point Likert scale: (1) increased, (2) stayed same, (3) decreased, and (4) don’t know or (1) a lot, (2) moderate amount, (3) not much, and (4) don’t know.

#### Key informant interviews

**Fig. 1:**
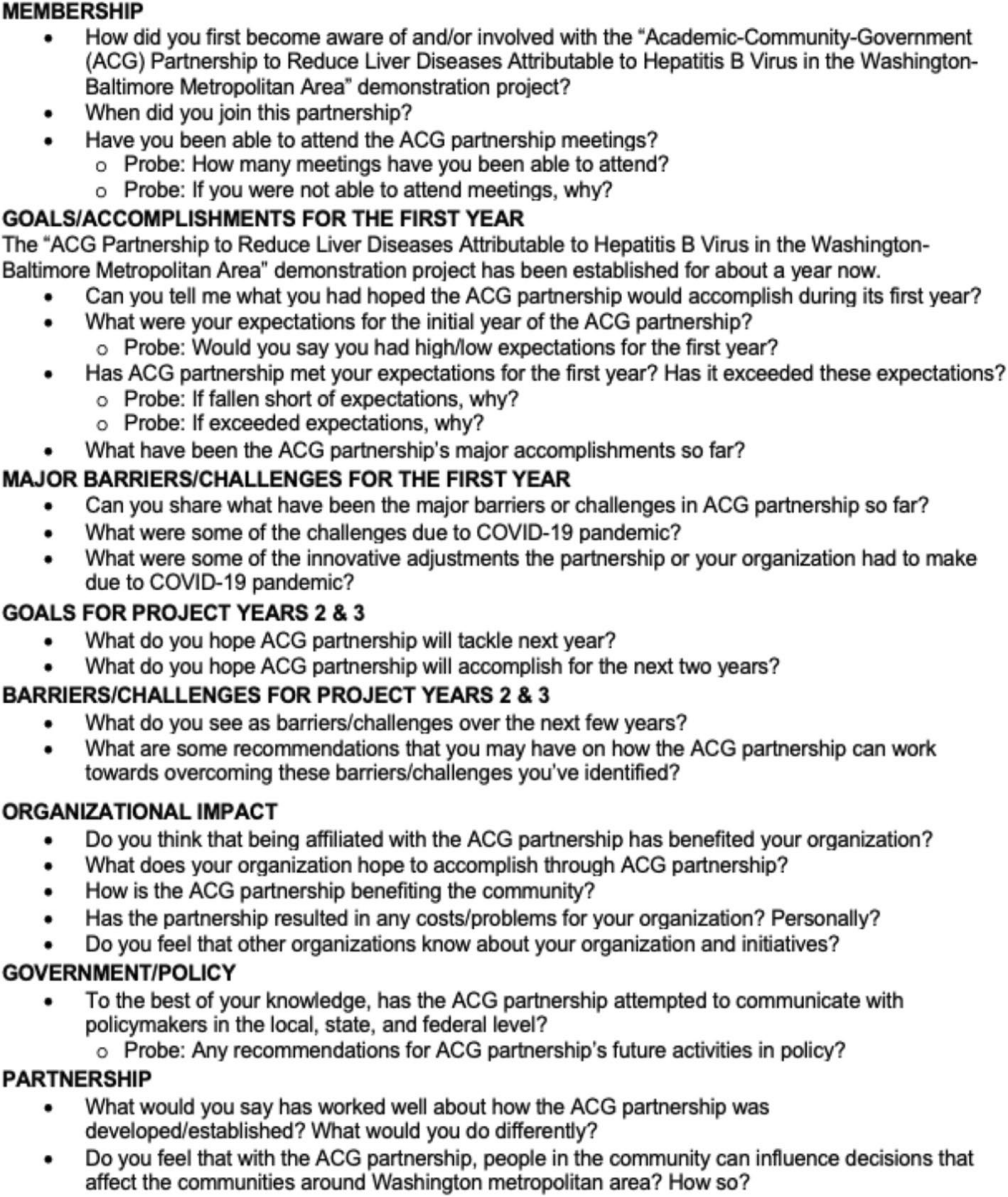
Interview guide

Upon survey completion, participating members of the WB-HBV Project Task Force were contacted by a graduate research assistant to schedule their subsequent follow-up key informant interview. The interview guide consisted of open-ended questions, also derived from Israel and colleagues [9], on capacity building of task force members to participate in CBPR partnerships (Fig. 1) [1, 3].

Interview questions focused primarily on core partnership content areas, which included membership, goals/accomplishments for the first year, major barriers/challenges for the first year, goals for Project Years 2 and 3, barriers/challenges for Project Years 2 and 3, organizational impact, government/policy, and partnership. Subtopics of further interest to the evaluation subcommittee included partnership history (e.g., role in partnership and governance; role of community members in the research process), co-learning experience (e.g., goals and barriers/limitations for Project Years 1, 2, and 3, benefits and challenges of working in the partnership), and perceptions of the ACG partnership’s effectiveness to address community needs. All interviews were conducted either via phone or WebEx by a trained research assistant and lasted no longer than 1 h.

#### Data analyses

Survey interview questions were administered online via Research Electronic Data Capture (REDCap), a secure web-based application designed to support data capture for research studies and hosted by the Clinical and Translational Science Institute at Children’s National [10]. The online survey data were downloaded into Statistical Package for the Social Sciences (SPSS) v.27.0, from which descriptive statistics (e.g., frequency distribution and comparison of means) were generated [11].

Audio-recorded interviews were transcribed verbatim (with members’ permission) for further review and analysis. Each transcript was thematically coded using QSR International’s NVivo software (version 12 Plus) [12] by at least two researchers and cross-checked for agreement. A taxonomy of emergent themes was developed and shared between all reviewers (DL, AC, MJJ, RH, AS) as an iterative and collaborative process. Content analysis was used to examine variations in the data to further refine the coding scheme [13, 14]. Themes were organized into overarching domains and compiled with representative quotations. Discrepancies were resolved through a process of constant comparison until inter-rater reliability was reached (Kappa coefficient > .80).

## Results

16 of 20 partnership members participated in the current study, completing either both the survey and the interview or at least one of these two components. There was one participant who completed the survey but not the interview, and there were two participants who completed the interview but not the survey.

### Results from the survey data

The survey was completed by 14 of 20 partnership members (70.0%): two academic, eight community, and four government members. Collaborative partnership members’ perspectives on partnership-building and research across the following 14 domains are presented in Table 1: organization and structure of meetings; trust; decisions; impact; general satisfaction; strategic planning; ACG policy impact; CBPR and government; participation in meetings; assessment of participation; partnership operations and capacity; communication; challenges/limitations associated with ACG involvement; and benefits compared to challenges associated with ACG involvement.

**Table 1:** Collaborative partnership members’ perspectives on partnership-building and research (*N* = 14)

	Total (M, SD)	Academic (M, SD)	Community (M, SD)	Government (M, SD)
**Organization and Structure of Meetings** (1) strongly agree, (2) agree, (3) neutral, (4) disagree, and (5) strongly disagree				
1. I find ACG partnership meetings useful.	1.71 (.726)	1.50 (.707)	1.88 (.835)	1.50 (.577)
2. The ACG partnership meetings are well organized.	2.14 (.770)	1.50 (.707)	2.38 (.744)	2.00 (.815)
3. Background materials (agendas, minutes, etc.) needed for meetings are prepared and distributed well in advance of meetings.	2.29 (.726)	2.50 (.707)	2.25 (.886)	2.25 (.500)
4. I wish we spent more time at partnership meetings hearing about and discussing ACG projects.	2.79 (.699)	3.00 (.000)	2.50 (.756)	3.25 (.500)
5. We do not accomplish very much at ACG partnership meetings.	3.57 (.756)	4.00 (1.414)	3.25 (.463)	4.00 (.816)
6. I believe that we adequately address all of the agenda items at the ACG meetings.	2.29 (.726)	1.50 (.707)	2.50 (.756)	2.25 (.500)
7. When I want to place something on the meeting agenda, I am comfortable with the process.	2.21 (.802)	1.50 (.707)	2.25 (.886)	2.50 (.577)
8. I would like more of a voice in determining agenda items for the ACG partnership meetings.	2.86 (.363)	3.00 (.000)	2.75 (.463)	3.00 (.000)
9. One person or group dominates at ACG partnership meetings.	3.50 (.760)	3.50 (.707)	3.25 (.707)	4.00 (.816)
**Trust—Part 1** (1) strongly agree, (2) agree, (3) neutral, (4) disagree, and (5) strongly disagree				
10. Relationships among ACG partnership members go beyond the individuals at the table to include member organizations.	2.21 (.893)	2.50 (2.121)	2.25 (.707)	2.00 (.816)
11. I am comfortable requesting assistance from other partnership members (when I feel that their input could be of value).	2.07 (.829)	1.50 (.707)	2.38 (.744)	1.75 (.957)
12. I can talk openly and honestly at the ACG partnership meetings.	2.14 (.770)	1.50 (.707)	2.25 (.886)	2.25 (.500)
13. I am comfortable bringing up new ideas at the ACG partnership meetings.	2.14 (.770)	1.50 (.707)	2.25 (.886)	2.25 (.500)
14. ACG partnership members respect each other’s point of view even if they might disagree.	2.29 (.611)	2.00 (.000)	2.38 (.744)	2.25 (.500)
15. My opinion is listened to and considered by other partnership members.	2.07 (.829)	1.50 (.707)	2.13 (.991)	2.25 (.500)
**Trust—Part 2** (1) increased, (2) stayed same, (3) decreased, and (4) don’t know				
16. In the past year, my willingness to speak and express my opinions at partnership meetings has:	2.31 (1.109)	1.50 (.707)	2.57 (1.134)	2.25 (1.258)
17. Over the past year, the amount of trust between ACG partnership members has:	2.29 (1.267)	2.50 (2.121)	2.63 (1.302)	1.50 (.577)
18. In the past year, the ACG partnership members’ capacity to work well together has:	2.14 (1.167)	1.50 (.707)	2.38 (1.188)	2.00 (1.414)
19. How much trust is there between partners now?	2.14 (1.351)	2.50 (2.121)	2.13 (1.356)	2.00 (1.414)
20. In the next year, how much trust do you expect to see between partners?	2.14 (1.351)	2.50 (2.121)	2.13 (1.356)	2.00 (1.414)
**Decisions** (1) strongly agree, (2) agree, (3) neutral, (4) disagree, and (5) strongly disagree				
21. I am satisfied with the overall way in which the ACG partnership makes decisions.	2.14 (.949)	2.00 (1.414)	2.13 (.991)	2.25 (.957)
22. All partnership members have a voice in decisions made by the group.	2.07 (.829)	1.50 (.707)	2.13 (.835)	2.25 (.957)
23. It often takes the ACG partnership too long to reach a decision.	2.86 (.770)	3.00 (.000)	2.75 (.886)	3.00 (.816)
**Impact** (1) strongly agree, (2) agree, (3) neutral, (4) disagree, and (5) strongly disagree				
24. The partnership of the ACG has been effective in achieving its goals.	2.00 (.784)	1.50 (.707)	2.13 (.835)	2.00 (.816)
25. The work of the ACG has brought benefits to my community.	2.07 (.917)	2.00 (1.414)	2.25 (.886)	1.75 (.957)
26. Participation in the ACG has increased my knowledge and understanding of the other organizations represented.	2.14 (.770)	2.00 (1.414)	2.25 (.707)	2.00 (.816)
27. Participation in the ACG has increased my knowledge of health disparities and social determinants of health.	2.00 (.784)	1.50 (.707)	2.00 (.926)	2.25 (.500)
28. Participation in the ACG has increased my organization’s capacity to conduct communitybased research.	2.14 (.864)	1.50 (.707)	2.25 (.886)	2.25 (.957)
29. ACG-affiliated projects are improving health outcomes for people in Washington DC metropolitan area.	1.86 (.864)	2.00 (1.414)	1.88 (.835)	1.75 (.957)
**General Satisfaction** (1) strongly agree, (2) agree, (3) neutral, (4) disagree, and (5) strongly disagree				
30. I am generally satisfied with the activities and progress of the ACG during the past year.	2.14 (.770)	2.00 (1.414)	2.25 (.707)	2.00 (.816)
31. I am satisfied with the types of projects that the ACG has implemented.	2.14 (.864)	2.00 (1.414)	2.38 (.744)	1.75 (.957)
32. I have adequate knowledge of the ACG budget, ACG resources, and how resources are allocated.	2.71 (1.139)	2.50 (2.121)	2.50 (1.069)	3.25 (.957)
33. I would like to have more input regarding the allocation of ACG resources.	2.79 (.699)	2.00 (1.414)	2.75 (.463)	3.25 (.500)
34. I am satisfied with the ACG’s efforts to translate research and evaluation results into information and programs that can improve health in Washington metropolitan area.	2.50 (.855)	2.00 (1.414)	2.75 (.886)	2.25 (.500)
35. I am satisfied with the ACG partnership’s attention to the ongoing sustainability of relationships within the partnership.	2.21 (.699)	2.00 (1.414)	2.25 (.707)	2.25 (.500)
36. I am satisfied with the ACG partnership’s attention to building the capacity of all partners to participate actively in the work of the partnership.	2.21 (.802)	2.00 (1.414)	2.38 (.744)	2.00 (.816)
**Strategic Planning** (1) strongly agree, (2) agree, (3) neutral, (4) disagree, and (5) strongly disagree				
37. Our strategic planning process resulted in the development of concrete goals and objectives.	2.29 (.726)	2.00 (1.414)	2.25 (.707)	2.50 (.577)
38. Our strategic planning process resulted in the development of appropriate strategies to accomplish our goals and objectives.	2.29 (.726)	2.00 (1.414)	2.25 (.707)	2.50 (.577)
39. Our strategic planning process has helped to sustain the ACG.	2.29 (.726)	2.00 (1.414)	2.25 (.707)	2.50 (.577)
**ACG Policy Impact** (1) strongly agree, (2) agree, (3) neutral, (4) disagree, and (5) strongly disagree				
40. The ACG has been effective in informing policymakers and key government officials about the ACG and its initiatives.	2.36 (.745)	2.00 (1.414)	2.38 (.744)	2.50 (.577)
41. Involvement with the ACG has provided support for policy issues my organization feels strongly about.	2.36 (.745)	2.00 (1.414)	2.38 (.744)	2.50 (.577)
42. The ACG has been effective at translating research findings into policy-relevant documents and educational materials.	2.57 (.852)	2.00 (1.414)	2.63 (.916)	2.75 (.500)
**CBPR and Government** (1) strongly agree, (2) agree, (3) neutral, (4) disagree, and (5) strongly disagree				
43. It is important that policymakers and key government officials are informed about the ACG and its initiatives.	1.50 (.650)	1.00 (.000)	1.63 (.744)	1.50 (.577)
44. Community interests are well represented in ACG activities.	2.14 (.949)	2.00 (1.414)	2.25 (.886)	2.00 (1.155)
45. I served as a co-presenter or presenter representing the ACG or one of its affiliated projects at a conference, training, or workshop/seminar.	2.64 (1.216)	2.50 (2.121)	2.63 (1.188)	2.75 (1.258)
**Participation in Meetings** (1) never, (2) 1–3 times, (3) 4–6 times, (4) 7–9 times, and (5) 10–11 times				
46. Please indicate approximately how many times over the last year you have attended ACG partnership meetings	2.14 (1.231)	4.00 (1.414)	1.63 (.744)	2.25 (1.258)
**Assessment of Participation** (1) strongly agree, (2) agree, (3) neutral, (4) disagree, and (5) strongly disagree				
47. I am satisfied with my level of participation in the ACG partnership.	2.29 (.825)	1.50 (.707)	2.38 (.916)	2.50 (.577)
48. I have taken advantage of opportunities to influence the work of the ACG partnership.	2.57 (.938)	2.50 (2.121)	2.38 (.916)	3.00 (.000)
49. I devote time outside of partnership meetings to ACG activities or projects.	2.64 (.842)	2.50 (2.121)	2.63 (.744)	2.75 (.500)
**Partnership Operations and Capacity** (1) strongly agree, (2) agree, (3) neutral, (4) disagree, and (5) strongly disagree				
50. The ACG partnership has a clear vision of what it aspires to achieve.	2.31 (.855)	2.31 (.855)	2.25 (.707)	2.33 (.577)
51. The ACG partnership vision has been translated into concrete, measurable goals that we aim to achieve.	2.23 (.725)	2.00 (1.414)	2.25 (.707)	2.33 (.577)
52. The ACG partnership effectively represents the diversity of our communities.	2.08 (.760)	1.50 (.707)	2.25 (.707)	2.00 (1.000)
53. Community interests are well represented in ACG activities.	2.00 (.816)	2.00 (1.414)	2.25 (.707)	1.33 (.577)
54. The ACG partnership thinks strategically.	2.08 (.760)	1.50 (.707)	2.38 (.744)	1.67 (.577)
55. The ACG partnership is well managed.	2.09 (.862)	2.00 (1.414)	2.25 (.886)	1.67 (.577)
56. The ACG is following its own CBPR principles.	2.15 (.987)	2.50 (2.121)	2.38 (.744)	1.33 (.577)
57. Partnership members take responsibility for getting work done.	1.85 (.801)	1.50 (.707)	2.13 (.835)	1.33 (.577)
58. In the past year, ACG partnership members’ capacity to work well together has increased.	1.92 (.954)	2.00 (1.414)	2.13 (.991)	1.33 (.577)
**Communication** (1) strongly agree, (2) agree, (3) neutral, (4) disagree, and (5) strongly disagree				
59. Members communicate effectively with each other during meetings.	1.85 (.801)	1.50 (.707)	2.13 (.835)	1.33 (.577)
60. Partnership members communicate effectively with each other outside of meetings.	1.92 (.862)	1.50 (.707)	2.13 (.835)	1.67 (1.155)
**Benefits of ACG Involvement** (1) strongly agree, (2) agree, (3) neutral, (4) disagree, and (5) strongly disagree				
61. Increasing recognition and respect for my organization in Washington metropolitan area.	2.25 (.866)	2.00 (1.414)	2.25 (.886)	2.50 (.707)
62. Developing new collaborative relationships between my organization and other ACG partner organizations.	1.92 (.900)	2.00 (1.414)	2.00 (.926)	1.50 (.707)
63. Working with communities with whom my organization has previously had little contact.	2.17 (.937)	2.00 (1.414)	1.50 (.707)	2.00 (1.414)
**Challenges/Limitations Associated with ACG Involvement** (1) strongly agree, (2) agree, (3) neutral, (4) disagree, and (5) strongly disagree				
64. ACG partnership activities do not address my organization’s goals and interests.	3.31 (1.109)	4.00 (1.414)	3.25 (.886)	3.00 (1.732)
65. Membership in the ACG partnership requires a considerable time commitment.	3.08 (.862)	3.50 (2.121)	2.75 (.463)	3.67(.577)
66. My (or my organization’s) opinion is not valued within the ACG partnership.	3.92 (1.038)	5.00 (.000)	3.38 (.916)	4.67 (.577)
67. There is too little funding for my organization’s participation in the ACG partnership.	3.46 (.967)	4.00 (1.414)	3.13 (.835)	4.00 (1.000)
**Benefits Compared to Challenges Associated with ACG Involvement** (1) yes vs. (2) no				
68. From your organization’s perspective, do the benefits of participation in the ACG partnership appear to outweigh the costs at this point?	1.27 (.467)	1.00 (.000)	1.17 (.408)	1.67 (.577)
69. From your personal perspective, do the benefits of participation in the ACG partnership appear to outweigh the costs at this point?	1.27 (.467)	1.00 (.000)	1.17 (.408)	1.67 (.577)

*M* Mean, *SD* Standard Deviation

#### Organization and structure of meetings

Members agreed that ACG partnership meetings were generally useful, well-prepared, and organized; many felt comfortable voicing their opinions at the meetings. Although individuals from the academic and government organizations felt that much was accomplished at these meetings and expressed neutrality regarding the statement, “I wish we spent more time at partnership meetings hearing about and discussing ACG projects,” community members expressed the need for increased dialogue but were neutral as to whether much was completed during the sessions.

#### Trust

Participants agreed that the relationships among ACG members extended to include member organizations beyond the individuals at the table. They expressed comfort in requesting assistance, introducing new ideas, speaking frankly, respecting each other’s viewpoints, and being heard. Academic members reflected a slight increase in comfort expressing opinions at meetings and confidence in ACG members collaborating; governmental members experienced increased trust between members over the year prior to the survey.

#### Decisions

While members expressed satisfaction with ACG decision-making and felt everyone contributed, they were neutral about the time the partnership took to reach decisions.

#### Impact

Participants felt the partnership was effective regarding its goals, benefits to the community, and improvements in health outcomes in the WBMA. They believed that participation increased understanding of the other organizations, knowledge on health disparities and the social determinants, and capacity for CBPR.

#### General satisfaction

Members were satisfied with the task force’s activities and progress, the types of projects implemented, attention to the sustainability of the partnership (as measured across the following three dimensions essential to sustainability: (1) relationships and commitments from partnership members, (2) knowledge, capacity, and values of the partnership, and (3) funding, staff, programs, policy changes, and partnership itself [15]), and capacity building of partners in the work. Community partners felt they had somewhat adequate knowledge of the ACG’s logistics and wanted to provide more input regarding the allocation of resources, while government partners were neutral. Community partners were less satisfied with the ACG’s translation of research and evaluation into information and programs to improve health in the WBMA.

#### Strategic planning

The sustainability of the ACG partnership depended on the task force’s strategic planning processes. Participants agreed that the task force’s strategic planning resulted in developing (1) concrete goals and objectives, and (2) appropriate strategies to accomplish them.

#### ACG policy impact

Participants felt the ACG was effective in informing policymakers about the ACG and its initiatives and in providing support for relevant policy issues. Community and government members felt that the ACG was less effective at translating findings into policy-relevant materials.

#### CBPR and government

Members strongly agreed on the importance of informing key government officials about the partnership and its initiatives. Members reported that community interests were well represented in ACG activities; however, opportunities to represent the ACG were somewhat limited for community members.

#### Participation in meetings

When asked how often participants attended ACG meetings over the last year, partnership members from the academic setting reported attending approximately seven to nine meetings; members from government and community reported attending only one to three meetings.

#### Assessment of participation

Most participants, especially academic members, indicated satisfaction with their participation level in the ACG partnership. Community members were more likely than their academic and government counterparts to believe that they influenced the work of the ACG partnership.

#### Partnership operations and capacity

All members agreed that the ACG partnership thought strategically, was well-managed, and had a clear vision of what it aspired to achieve, and that the vision had concrete, measurable goals that members aimed to achieve. Members also felt that the ACG follows its own CBPR principles, effectively represents the diversity of the communities, and represents community interests in ACG activities. However, academic partners, compared to other members, differed on whether the ACG partnership has a clear vision of what it aspires to achieve and follows its own CBPR principles. Government members were more likely to respond favorably to the majority of the partnership operations and capacity measures. Across the nine measures within this domain, participants most enthusiastically agreed that ACG partnership members took responsibility for getting the work done, and their capacity to work well together had increased in the past year.

#### Communication

Participants agreed that ACG members effectively communicated with one another during and outside of partnership meetings; members from the academic and government settings, however, expressed slightly stronger agreement than their counterparts from the community.

#### Benefits of ACG involvement

Participants unanimously agreed that various benefits were gained from the ACG partnership. Academic members were more likely than their partnership colleagues to agree that “an increase in recognition and respect for their institution in the WBMA” occurred, while community and government members were more likely to report “opportunities to work with communities with whom the partnering organizations have previously had minimal contact with” and “development of new collaborative relationships across the partnering organizations” as benefits of their ACG involvement, respectively.

#### Challenges/limitations (associated with ACG involvement)

Academic members agreed that the ACG partnership activities addressed their overall goals and interests, while community and government members’ feelings on this topic remained more neutral. Although academic and government members were more likely to agree that their opinions (and their organizations’ opinions) were valued, and substantial funding existed for their respective organizations to participate in the partnership, community partners continued to express neutrality in their responses on these measures.

#### Benefits compared to challenges (associated with ACG involvement)

From organizational and personal perspectives, academic and community members were more likely than their government counterparts to agree that the benefits of participating in the ACG partnership outweighed any challenges encountered to date.

### Results from the in-depth interviews

Qualitative interviews were conducted with 15 of the 20 partnership members (75.0%): two academic, nine community, and four government members. Interviews lasted from 30 to 45 minutes. As presented in Table 2, four main themes were identified relating to the ACG partnership: (1) partnership involvement, (2) project goals and accomplishments, (3) project challenges and barriers, and (4) partnership’s involvement in government or policy.

**Table 2:** Themes and codes

Theme	Code	Representative Quote
**Partnership involvement**	**Benefits of the ACG partnership**	**Synergistic Partnership With current project goals, the community partners are now contacting other communities in need and not just the population previously served:** • “So we’re able to actually tap into a Hispanic group. We also did some LGBT (lesbian, gay, bisexual, and transgender) groups, as well. And we also got into the people, PWID (people who inject drugs), so we actually get to the community as well. So with that project that actually helped us expand even further out.” (community partner) **Three organizations working together for a common goal—betterment of the community:** • “But what I do see is most of these agencies and centers never interact with the academic community. And I think this partnership is showing them or at least making them aware of the fact that the universities also see this as a problem, hepatitis B in the U.S., so I think from that end, it probably makes them feel good that they’re not the only ones who are saying this is a problem. But the universities with the big hospitals also see that’s an issue.” (community partner) • “[…] actually bring a large segment of different areas of service of public health service to the table is a big accomplishment and a big positive.” (government member) **New Knowledge** **Partnership members are gaining new knowledge regarding HBV throughout this partnership project:** • “I think we’ve learned a lot, or there’s an opportunity to learn a lot in terms of where the gaps are, what kind of things are needed to improve services for this population.” (academic member) • “Work with the partnership has increased my awareness of HBV, my understanding of the disease and of the morbidity, mortality, statistics, and how it impacts especially our Asian immigrant and African immigrant communities, and Hispanics, not only here […] but nationally.” (community partner) **Organizations have expanded their network due to current partnership:** • “So just trying to work closely with them to better understand their networks, and how we can leverage that, in order for all of us to just be in communication and helping one another and spreading resources for the patients to reduce liver disease.” (government member) **The partnership is working together, making small incremental steps to reduce disparities for better community health:** • “So it’s small, incremental things, making positive, incremental changes, that’s what we’ve been doing. And I think what’s reflected on the community, it may not be overnight, but you can see the impact over time.” (academic member)
**ACG partnership project goals and accomplishments**	**Year 1 Goals**	**Goals for ACG partnership** **To achieve the goals of ACG partnership and increase communication between** **partners:** • “My expectation is to achieve all our goals, and we work towards achieving the goals and hopefully to exceed as well. […] I was expecting to have better communications.” (community partner) **Quarterly reports** • “Every quarter, we have a quarterly report.” (academic member) **HBV-related Goals** **Education and linkage to care:** • “We hope to during the first year… educate more people about the prevalence and have hepatitis B, and then we hope to connect them also with resources that are in the communities.” (community partner) **Screening:** • “[…] getting more people to screen […] We need more funding for more screening, more outreach.” (community partner) **Vaccination:** • “They were trying to see if they can increase the number of people who were vaccinated, those coming from the high endemic areas of hepatitis B, etc., and also increase the vaccination and follow up for pregnant women and children.” (community partner) **Resources:** • “Resources as, like I said, a lot of our patients don’t have insurance. So they …have to pay out of pocket to see a doctor. But even you know, most of our patients can’t afford that. And then on top of that, if they’re positive and their viral load is high, they need medication. And unfortunately, you know, hepatitis B has no cure. So they’re going to be on medication and monitoring their entire life. So a lot of people with that extra expense, they’re not going to be compliant with medication and getting checked every six months. So resources would be providers that see patients just for free, or medication programs for free. … So location, transportation also falls into that category [of] resources. And then the last thing is vaccination because the Asian community has a higher risk of HBV, we usually recommend that they [and] their family get vaccinated.” (community partner) **Not sure of goals:** • “I don’t know if they didn’t get or not.” (government member)
	**Accomplishments so far**	**HBV-related accomplishments** **Exceeded objectives:** • “We exceeded all our deliverables. And even during this pandemic.” (community partner) **Related to COVID-19:** • “Especially with this year, COVID, there’s had to be a lot of adjustments made in terms of how to effectively reach the target population, and keep them safe.” (community partner) **ACG partnership goals** **Building capacity:** • “I think things happen along the continuum. I think that we have made some progress in terms of building capacity, in terms of engaging the community, but I think the real impact is not there yet.” (academic member) **Funding and leadership:** • “And that’s funding […] and with the leadership […] has been very successful around funding.” (community partner) **Presentations:** • “All the presentations […] at least four or five on the first years and completing, finishing all the other reports quarterly report.” (academic member) **Having everyone at the table:** • “Being able to pull all the partners together at the table is definitely part of the milestone to get people or organizations involved. So, that part? Yes. The details of the execution, I think is an ongoing.” (community partner) • “When they do test positive, there’s about 4 to 5% who test positive for B and about 2 or 3% for C, and all those individuals are contacted and linked to care services. And so right now, I think there’s a little bit more effort being put into following up with those individuals to see whether or not they are actually seeking treatment and on schedule for treatment.” (community partner) • “I think that the way in which they were able to bring a mix of academia, direct service providers, and government to the table, it’s always progress. And this is a major step towards doing much larger to have an impact on community health. I think that that was a terrific approach. And clearly, they weren’t doing that. Clearly they weren’t achieving that. But we were all at the table.” (government member) **Others:** • “I think it accomplished that particular goal. And that’s to take a program that’s on paper and to operationalize it. And to have good reporting systems, have a good relationship between the partners, which the program has. There’s excellent relationships between the partners. Good data. So I think it accomplished having the data. And I think more importantly than all of that, well, maybe as a result of all of that, what I think we all were able to, to actually to actualize was to be considered probably the best program in the country, among the five or six demonstration programs that were funded over a year ago with the system’s good data.” (community partner)
	**Future goals for screening**	**Sustainability of screening and vaccination:** • “We need to work towards the sustainability of the screening for the hepatitis screening either at the clinics or private doctor’s offices. And then the vaccination as well.” (community partner) **Involving “champions” in the community:** • “What I hope to tackle next year is to be able to build around us people like champions in the community that will be able to multiply what we do in one place to other places.” (community partner) **Birth dose:** • “Most of the clinics, and even the centers that are in this partnership do not work directly with pregnant women. And I think that’s one of the requests of the grant. So hopefully, they’ll find a way of incorporating that in the second and third year.” (community partner) **COVID-19:** • “I think it’s also going to depend on this vaccination for COVID-19. And how successful that would be because then people will then start feeling comfortable to come into the clinics and the centers. And then the providers also feel comfortable going out to meet people to do the work. So, but I think they’re doing a really, really good job.” (community partner) **Working with other community organizations, churches:** • “As we all know, the people from the endemic areas are mostly people who are very religious, and the churches are still open. So if we’re able to access the churches and do trainings in the churches, we’ll still be able to reach the target population.” (community member) **Educating community about COVID-19:** • “[…] helping to educate the population about COVID.” (academic member) **Screening, educating, vaccinating:** • “Just screen as many patients as we can, provide education, more vaccines to prevent HBV and get people treated and linked if they test positive.” (community member) **Health Fair:** • “So we are planning a health fair […] So we have more people vaccinate, and we have more people come out for the health fair, and it’s always a good way for us to boost up the number.” (community member)
	**Future goals for ACG partnership and recommendations**	**Goals for systems** **Continuity of care:** • “The first area has to do with how well governmental partners such as the health departments work together with the community partners, so that there’s much more of an ability to have continuity, especially for those persons who are HBV positive, helping to make sure that the reporting systems between community and governmental entities are strengthened. Also, so that’s much more of your health information exchange goal that’s in the proposal. So I’m hoping that in Year Two and Year Three that gets attention.” (community member) **EMR, standardizing screening:** • “There is a goal related to electronic medical records so that HBV becomes a part of anyone’s care coming in through the door. […] So standardizing HBV screening and care as part of patient care is important.” (community member) **Raising awareness about HBV among minorities:** • “Raising awareness, letting people know that there’s a lot of communities like Asians and Africans, where HBV is prevalent, and it’s not just because of STDs, but it’s because of where these are from. So that people are aware that there are big groups of hepatitis B positive patients that need to be addressed. So that we aren’t spreading.” (community member) **Dissemination:** • “There could be a summit or some sort of conference. There could be a creation of some sort of case consultation, for example, around perinatal hepatitis B. There could be engagement with medical providers around hepatitis B as an issue. There could be a coordinated activity where maybe one day is selected to actually promote and provide, for example, hepatitis B-related services. Whether it’s general community education, or otherwise, an emphasis on testing, for example, but that it would happen in multiple sites at the same time and promoted by all as one group one body.” (government member) **Refine Project in the context of COVID-19 pandemic:** • “We need to now begin to think in the context of implementing this program. In the context of COVID… COVID will still be with us next year. So the issue of safety precautions and ensuring implementation of community testing and community events in a safe manner will still be very relevant, so that context needs to be built into the program. […] Nobody was thinking of COVID before. Implementing this kind of program, this academic, community, and government partnership project to reduce hepatitis B virus, needs to be built with a strong context around COVID-19 in mind.” (government member) **Other objectives** **Birth dose:** • “Another thing came up more recently is the birth dose, Hep B birth dose […], engagement, and tracking and that kind of things. And mostly previously, it’s more on immigrants - African and Asian. So we need to figure out how to engage with pregnant women and get into vaccinating when they have a baby within a day. So that would be one thing we hope to accomplish with documented changes and interventions and see we can get something done.” (academic member) **Unsure** **Unsure about goals for Years 2 and 3:** • “That I’m not sure.” (community member) **Need for clear communication and attendance in meetings to better understand project goals:** • “We weren’t included in more of those meetings that may help us better understand other partners who are involved in other parties that we can leverage or who may be interested in working with us.” (government member) • “If we were to attend more meetings, I definitely think that could be advantageous to us.” (government member) • “And so what I really do hope for the project is that they continue to work together as a partnership. And really solidify that partnership and a couple of areas that are in the grant proposal that still needs strengthening.” (community member) • “Hopefully, we can have more engagement, communication, a meeting. So currently, for example, just some of us meeting more regularly. Hopefully will be more frequent. But this could be a downside to that. And just more time will be involved.” (academic member)
**ACG partnership project challenges and barriers**	**Overall challenges so far**	**ACG partnership** **Time to communicate:** • “It’s a major barrier and challenges with the partnership. It’s coordinating the time to communicate. Coordinating and scheduling time to coordinate. […] And we were planning to do three meetings for Year One. But the third meeting, we’re not able to do because of COVID, and that’s our biggest challenge.” (community member) **Addressing birth dose:** • “One of the challenges is that one of the goals was to focus on birth dose and trying to identify the best way to look at that, measure that, examine that. So that’s been a challenge. And so I think the team has been really working hard trying to identify ways they could address birth dose.” (academic member) **Being clear on goals and outcomes:** • “One challenge, again, is that I think the whole partnership needs to be clear on what the overall goals are, and what the outcomes are. So that has not been as transparent as I think I would like.” (academic member) **HBV** **Reaching out to target population:** • “The barriers during the first year I think it was basically to get the word around and then get the people we found positive to get treatment.” (community member) **Lack of ability to reach out to the community and other racial/ethnic minorities:** • “What we lack is the ability to reach out to the community and other ethnicity organizations.” (community member) **Mistrust from community:** • “We had to learn ways to adapt. […] What are you here for, there’s no such thing as free. So that’s always a barrier, is there cost or the hidden costs?” (community member) **Working with three different communities:** • “We have to be cognizant that each of these has their own dynamic within their community, how they are seeing, who they connect with, what, how they can work with their policymakers, so on and so forth, how they work within their medical and health, infrastructure within that community. I think that’s the part that may be lacking a bit. How do we build up each of our partners within the networks that they live within?” (community member)
	**Anticipated future challenges and barriers and recommendations to overcome challenges**	**Related to HBV** **Continued challenges due to COVID-19:** • “The challenges so far remain just the COVID issue. […] And we hoped that it can get in control as soon as possible so we can go back to those large physical settings that we use to do in order to educate more people.” (community member) **Related to ACG Partnership** **Lack of funding:** • “I think funding, funding from CDC is a major barrier and that needs to be brought to their attention.” (community member) • “Funding is usually a major barrier.” (community member) **Transparency of partnership goals and outcomes:** • “One challenge, again, is that I think the whole partnership needs to be clear on what the overall goals are, and what the outcomes are. So that has not been as transparent as I think I would like.” (academic member) **Equitable relationship with partners:** • “I would make sure that the community partner, their relationship is equitable, that it is not so academic driven.” (academic member) **Challenges in Health Information Exchange:** • “Building up that capacity around EMR systems, I think that’s going to go on for a while. And hopefully, we can get that together so that it becomes standardized practice.” (community member) **Challenges in evaluation and sustainability of the program:** • “So I do think that heading toward the middle of Year Two and Year Three, there has to be a conversation about the partnership, whether it’s a partnership that’s going to disappear, or whether there are monies that hold the partnership together, or even without money as what other things do they have in common that really motivates them to stay together as a partnership. That’s going to be a challenge.” (community member) **Completion of the project:** • “So we have one year, a few months under our belt. And I think one challenge …, we have to be more open to different audiences, different settings, and engage with different populations. So that could be a challenge as well. So that’s why we hope for completion but the challenge for us is the pandemic.” (academic member) **Time commitment:** • “In the perspective of managing the national task force and hepatitis B, we have monthly meetings. And I think one of the biggest challenges of having standing meetings is the time to time commitment, especially, if you’re asking individuals who are not doing this as a full-time job, to carve out some time during the workweek to meet, I think that could be potentially one of the biggest challenges if this is not their full-time job.” (community member)
**ACG partnership involvement in government or policy**	**Current involvement in government or policy**	**Current involvement in government or policy:** • “Your local department of health is sort of like your policy agency, in many respects, at least for local policy. So the fact that we’ve been at the table would sort of lead to that.” (government member) **Not currently involved in government or policy:** • “We are so busy doing the groundwork, doing the grassroots work. So we never have an opportunity to talk to the government or the academic.” (community member) • “I don’t think this project is involved in any of the policy.” (community member) **Uncertain:** • “I believe so. So I myself didn’t really pay a lot of attention on the policy level, because [I am] occupied with tasks.” (community member) • “I’m not sure about this answer, but I will, I would think yes, but I’m not sure about this answer.” (community member) • “To the best of my knowledge, I have no idea. I really don’t.” (community member)
	**Future recommendations for involvement in government or policy**	**Increase Community Testing/Screening, Vaccination, and Education** **Increase testing available to minority population:** • “Hepatitis B vaccination should be made available free for people who don’t have insurance, especially for the adult population.” (community partner) **Immigrant Health Policy** **Dissemination of findings to legislatures interested in immigrant health:** • “In the Chinese community, the medical community, there are professionals trying to advocate for the community for hepatitis B, hepatitis B resource. So I know, at least like 10 years ago, a physician, […] he was able to really go to the Capitol Hill and fascinate the Congress. So we have an event like that. And he was very successful.” (community partner)

#### Partnership involvement

##### Role and involvement

Community partners were tasked to screen individuals for HBV surface antibodies, HBV surface antigens, and HBV core antibodies. Among vaccinated individuals, community partners were also tasked to confirm which HBV vaccine was administered - either the 2-dose Heplisev, the 3-dose Engerix B, or the Recombivax HB. Health department partners were expected to help recruit participants, interpret data collected, and support the partnership activities. Academic partners were tasked to complete IRB application and protocol development as well as manage all community partner data collection, aggregation, and reporting to the federal funder. In addition, evaluation teams were expected to evaluate process and outcome measures.

##### Benefits of the ACG Partnership

Participants described multiple benefits of the ACG partnership. The primary benefits described included the synergistic relationship of the organizations to better address HBV disparities in the WBMA community and the increased communication with other organizations due to partnership. A community organization member said:

So this partnership is so crucial, you know, it needs, like this table needs four legs, and it needs this four legs in order for it to work... and so being in this project, it has actually created this big impact. Because, you know, now we can communicate with the Department of Health, we can communicate with the academics, you know, to do publication, so more people are gonna be aware. And so, so the impact is big.

Another policy benefit mentioned was the partnership’s ability to inform policy through information provided to the academic and government organizations. A community member stated, “It’s important to be able to report that information and share it in the academic world. And that those reports and research findings are also shared with the government so they can make better policy decisions.” Moreover, through the partnership, community organizations can work with the academic and government organizations to more efficiently and effectively reach individuals who need help, execute project plans, and disseminate research findings beyond the academic and government settings and into the communities. As two community members pointed out:

Partnerships are to strengthen community relationships and your ability to outreach to the target population, so that actually gave us a better understanding of actually, what ethnicity needs more help. Back then we only mainly focused on the Asian, and African; now we go into the Black community, the LGBT group, and people [who use] drugs.

It’s benefiting them in the sense that they have somebody and people that are well, knowledgeable about things about issues that affect the community, they are benefiting about the fact that we bring to the community and close to them, you know, services and resources that they can use in order to continue to get in control of their own health.

In addition, partnership members affirmed that funding has helped with resources, especially at the start of the Coronavirus 2019 (COVID-19) pandemic, when people were losing jobs and a lot of attention was paid to infectious disease screening and vaccinations. An academic member said, “Several people got funded and other groups as well through this grant during the pandemic. I think it’s really helpful for them. And also, through this grant, more people are getting screened and vaccinated, and linked to care. So that’s, I think it’s substantial health benefits as well.”

#### Project goals and accomplishments

##### Year 1 goals

Several goals were mentioned by partnership members, including goals within the ACG partnership and HBV-specific goals. Within the partnership, members wanted to “have more communications,” build capacity, and complete all contractual deliverables. In terms of building capacity, a community member highlighted the importance of having multiple perspectives in the planning and delivery of services:

Yeah, so my understanding when the partnership meetings were set up, it was to engage different stakeholders in the decision-making of being able to outreach, educate, screen link patients to care services relating to hepatitis B, and possibly C, depending on the stakeholders and, you know, where their focus and interest is. So, the stakeholders should have included community-based organizations, the academic institutes, and the government agency, so that there are multifaceted perspectives in the decision-making of that cascade of care.

Regarding goals related to HBV, participants indicated increasing education, screening, vaccination, outreach, resources, and linkage to care. Community members conveyed that providing education and resources and linking patients to care will help lower infections and “bring awareness to the disparities and the different communities that have HBV, and ultimately … a little bit more resources to help these patients.”

Although some members knew the screening goals, others were not too certain what the task force’s project goals were. One commented, “So I’m not really sure what the overall goals were and if they met them or not.” Recommendations included further strengthening the communication between core members of the ACG leadership team (specifically on what the future goals for the partnership should be) and increasing overall engagement and attendance at the ACG meetings.

##### Accomplishments to-date

Members confirmed that the ACG partnership exceeded their goals; some expressed their appreciation for how quickly team protocols were adjusted to keep participants safe at the start of the COVID-19 pandemic. Screening brought opportunities to link individuals who tested positive to care and vaccinate individuals who tested negative. For example, one community member remarked, “Screening more patients, obviously finding those positives and then I guess for the people that tested negative but who are not immune, then they can get a chance of getting vaccinated and protecting themselves. So those are the things that we were able to accomplish.” Especially for minorities without insurance, this project was important in increasing the overall HBV vaccination rate. One member from a government organization stated, “We also help to improve access to hepatitis B vaccination. We can provide hepatitis B vaccination for adults who are uninsured. And this is a critical population, especially among immigrants new to the country, [who] don’t have insurance yet.” One of the community partnership members further elaborated on how hepatitis prevention is not a priority to the immigrant population: “They have a lot of priority to deal with. So prevention care is definitely not in priority.” Thus, the ACG partnership is taking a big step forward in preventing hepatitis in the immigrant population.

In terms of the partnership, members expressed that the organizations have worked well together by having everyone at the table share, communicate strategies, build capacity, and engage the community. One participant from a government organization emphasized that these types of partnerships can help build future partnerships and collaboration among different organizations working toward a common goal. One partnership member mentioned how obtaining funding was particularly successful in the past year. Another government member discussed how sharing project updates and strategies to overcome barriers was especially helpful:

They give progress reports, they disclose challenges to implementation. I remember, the last one, we talked a lot about our COVID as it impacted the community outreach, testing, and ways that they can go around the outcome, we continue to make sure that we’re providing services, even amid COVID intervention, I think this is really very good.

##### Future goals for screening

Partnership member goals included meeting set numbers for screening, increasing education and vaccination, having sustainability of screening and vaccination, involving “champions in the community,” addressing HBV birth dose, working with community organizations such as churches, educating the community about COVID-19 as partners adjust to best accommodate patients, and raising awareness about the hybrid model so members can register online to get tested. For example, a community member shared, “I mean, just maybe raise more awareness and let people know that [we have] that hybrid screening model so that, you know, if they’re uncomfortable coming up to a group setting, they can always register online or let them let other people know about our online registration to get tested.” Another participant hoped for a health fair to increase the number of people screened and vaccinated for HBV.

##### Future goals and recommendations for the ACG Partnership

Goals for the ACG partnership include raising awareness about the high prevalence of HBV among Asian and African immigrants and receiving continued funding from government entities. Two members from community and government organizations (respectively) shared:

I think, more importantly, is rais [ing] awareness with the government agency that’s providing the funding to make sure that funding continues because of the important work that’s being done in the unmet needs that’s happening in viral hepatitis world, especially with hepatitis B. So I think, you know, in that coalition meeting, what needs to be addressed is the understanding that this is still an ongoing disease burden that needs attention... and making sure that, you know, there, there is a sustainable plan, in terms of helping patients realize what the disease burden is, and to raise awareness for prevention.

There might be an opportunity [to] enhance support or funding for hepatitis B. That, I think, would be necessary. And it’s only just now happening for hepatitis C. And this is very recent. I think hepatitis B needs to be looked at similarly.

Other objectives include providing continuity of care for individuals who test HBV positive, strengthening reporting systems and health information exchanges using electronic medical records (EMRs), and addressing HBV as standardized screening. One community member shared:

That’s an important goal, having the EMR systems that are able to do that are fundamental to that process, because it helps clinicians, as they log on, to always be reminded that HBV is part of routine patient care. So that in Year 2 and Year 3, I hope that there’s work to strengthen the EMR component, as well as the health information exchange component, so that both the internal work around HBV and the internal to external reporting and management are both strengthened.

#### Project challenges and barriers

##### Overall challenges to-date

Members revealed that coordinating project meeting times has been the biggest challenge, especially for those not working full-time. As one community member shared:

I think one of the biggest challenges of having standing meetings is the time commitment, especially if you’re asking individuals who are not doing this as a full-time job to carve out some time during the work week to meet, I think that could be potentially one of the biggest challenges if this is not their full-time job.

Contrary to their colleagues in the partnership, community members heavily emphasized “how [their] membership on the WB-HBV Project Task Force required/s a considerable time commitment.” Attendance at meetings by community members (1–3 meetings) was considerably lower than attendance by academics (7–9 meetings). Some participants cited “busyness”, “being overly committed with other responsibilities”, and “low staff coverage to attend partnership meetings” as possible reasons. As our project standing meetings were originally agreed upon when the partnership initially formed, pre-covid, it is very likely that new/additional responsibilities naturally arose over time due to the competing/immediate priorities caused by the pandemic (especially for our community partners who were active “in the field”).

In terms of communication between partners, members initially were not made aware of the expectations and did not clearly understand project goals and outcomes. However, once information was shared with them, they had a better grasp on their involvement and contribution to the partnership. For example, one community member revealed:

In the beginning, we were having trouble kind of just gathering all our information because everything just started so we didn’t really know what information was expected from our organization, like number-wise and stuff like that. But once we kind of understood what was expected [it] was easier.

For HBV-related activities (screening, linkage to care, and vaccination), some of the overall challenges mentioned included contacting the target population (including local organizations that serve racial/ethnic minority communities), linking them to care, and working with different communities in the WBMA area. A community member described mistrust as a challenge in HBV-related activities because community organizations are viewed differently from hospitals:

When they come, and of course, we have our banner, our website, and what we offer, but to them, it’s still, “You’re not a hospital. So why should we go there and get our blood drawn [ … ] from someone we don’t know?”

##### Anticipated future challenges and barriers and recommendations to overcome challenges

Several anticipated challenges were presented by partnership members. One of the biggest challenges was associated with COVID-19, which affected in-person interactions, and therefore limited screening events, community health fairs, and patients’ access to care. A community member shared, “So they’re still not comfortable and that is kind of still a challenge because every year we used to have, like, really big health fairs whereby we are present in person, you know. Anything that you do online is very different from what you have in person.” Members also anticipated challenges to reaching other racial/ethnic minorities communities, such as the Latino or Hispanic populations. Although they have identified these as priority populations, the partnership has not yet established relationships with organizations that predominantly serve these populations. One community member said, “So that was the part that’s new to us, the Hispanic community. We’ve never been able to get into that community. [ …] So that’s what we need, to get into that community more. So I think that’s our barrier.”

Since HBV is incurable and requires prevention resources, challenges within the ACG partnership may include a lack of funding for screening and education or overall funding for HBV-related activities. One member from a government organization stated:

For example, there might be an opportunity to enhance support for funding for hepatitis B. That, I think, would be necessary. And it’s only just now happening for hepatitis C. And this is very recent. I think hepatitis B needs to be looked at similarly. It’s always included, but it’s never received the right attention because it’s not curable.

Another community member mentioned that using evaluation to inform programming, sustain HBV-related activities or programs, and build capacity around health information exchanges or EMRs will be important future work of the ACG partnership that will allow for “a stronger linkage between the community, the community partners and the health department around data.” Members also emphasized that important factors for the ACG partnership are clear communication and transparent goals and outcomes, equitable relationships with partners, and commitment of time.

#### Partnership’s involvement in government or policy

##### Current involvement in government or policy

Overall, partnership members indicated that more work should be done to increase HBV funding. One community member shared:

I think there should be more work done with state legislators as well as state viral hepatitis coordinators. But it all really comes down to the federal government and whether or not there’s the budget allocated to address hepatitis B in particular, because I know there is already funding for hepatitis C.

##### Future recommendations for involvement in government or policy

Partnership members felt that future efforts should include increasing education and HBV vaccine availability and addressing vaccine hesitancy. A community member noted that testing should be widely available to minority populations through similar projects:

Yes, my recommendation is to continue to increase the number of testing, having testing available to help the minority population to get to the point of care. So, if they can have more testing and more partnerships in [the] community that will be a good thing. [Especially] if the work that is being done now is [replicated] to have more people and more partners so that while we are targeting one community, other communities [are] being targeted somewhere else.

In addition, several members emphasized the importance of disseminating findings of the project to legislatures interested in immigrant health, which has been successfully done in the past. One member from the academic organization stated, “I think one thing would be having speakers come in, having us do a presentation with the legislator … and I think when we talk about the dissemination of the findings, making sure we disseminate our findings back to legislators, as they are key stakeholders.”

## Discussion

This study provides insight into the facilitators and challenges of ACG partnerships and the importance of conducting partnership evaluations to improve partnership function. The process evaluation of the WB-HBV Demonstration Project highlights the strengths of an ACG partnership in addressing HBV disparities among racial/ethnic minorities and at-risk communities [16]. Most notably, during their first year, the partnership was able to reach its goal of screening 2300 persons by screening 2495 persons, and provided 408 vaccine doses, of its projected goal of 400 doses. Overall, ACG members highlighted the partnership’s positive impact at informing HBV-related policy, reaching project goals and objectives, and overcoming barriers with innovative solutions during the COVID-19 pandemic. Partnership members also described challenges and areas to improve including communicating clear goals and outcomes, task force meetings that are attended by everyone, and overcoming COVID-19-related difficulties in reaching the target population.

Different organizations provided a variety of feedback related to participation: community organization members felt more strongly that they had a positive impact on the partnership, expressed a strong desire to contribute more in the allocation of resources, and described the time required to participate as a major challenge; academic members attended far more meetings (seven to nine versus one to three for other members), felt the most satisfied with their participation, but were more varied on the clarity of the partnership’s vision; government members were the most positive about the partnership’s clear vision and following its own principles, and (with communities members) were least positive about the policy-impacting materials created as a result of the partnership. These variations suggest that members of different organizations have different experiences in, and expectations for, the ACG partnership and introduce the question of how best to structure partnerships so they are more equitable for the members.

The existing literature from recent process evaluations of CBPR partnerships validates (a) the value of the CBPR process for improved outcomes; (b) the importance of community members being involved at every stage of the process; (c) the importance of relationship- and trust-building with community organizations; and (d) the importance of the community to take ownership of the project [17–23]. The literature also highlights the challenges of communication, inclusiveness, and community involvement to successful CBPR [17–25].

CBPR, which necessitates an active partnership of equal commitment among various organizations throughout the entire research process, is essential in addressing health disparities such as HBV among racial/ethnic minority populations [26]. CBPR has been long implemented in other communities such as the highly noted Detroit Community-Academic Urban Research Center’s Neighborhoods Working in Partnership (NWP) [1]. In this current study, partners contributed their own expertise and worked together to improve the lives of community members living daily with HBV [1]. Similarly, for this current partnership, all members of the project team were involved in the process evaluation, which was a method used to assess the process, impact, and effectiveness of the WB-HBV Project. Findings strongly highlighted the importance for partners to clearly (1) communicate and delineate the value of the partnership and meeting structure and (2) convey the important role and contributions that community participants play and provide. It is especially critical, at the early stages, for ACG partnerships to create shared visions, norms, and equitable leadership roles for all agencies/organizations involved [27]. This helps to create buy-in to the collaboration and the partnership, and also motivation to contribute in terms of time and commitment including attending meetings. Often it is the academic partners doing the scheduling and attention needs to be given allowing the community members to do the scheduling of events and meeting with assistance from the academic partners for equitability [27, 28]. Commitment and investment of time can further be established by creating bi-directional pathways of learning; it is just as important that the ACG partnership that sets this also has a priority to elicit investments of time, attention, and attendance to the partnership [29].

The outcomes of our process evaluation comparably reflect those as described in Israel et al. (2010) [1], and were used to inform future CBPR efforts needed in DC communities to enhance capacity building and advocate policy changes. A previous process evaluation of CBPR in addressing HBV disparities highlighted that ongoing process evaluations are necessary to assess various partners’ current work, goals, and objectives and, ultimately, to achieve stronger cohesion in reaching partnership goals [30]. Specifically, there are several strategies and techniques that can increase the likelihood of partnership success [31–35]. As identified by Israel and colleagues [15, 23, 31], these should include (1) the facilitation of collaborative partnerships in all phases of the research, (2) the integration of knowledge and action for mutual benefit of all partners, and (3) the promotion a co-learning and empowering process that attends to social inequalities. Recognition of and responsiveness to group dynamics [31, 34, 35], including the shared development of clear and operational group goals that emphasize cooperation but reflect individual interests, contributes to the effectiveness of a diverse collaborating group, as does a climate that supports group cohesion [31–34]. In order for agreeable project aims and outcome expectations to be shared by partnership members, specific activities such as determining goals and prioritizing tasks based on theoretical frameworks [23], adopting a centralized communication network where a few members receive and share information [31], and encouraging small-group work within each organization to identify any issues and/or to regularly solicit ideas [31] are also necessary to allow for members to have a sense of group belonging and project accountability through team connectivity. Processes that facilitate equitably-distributed participation, open communication, and leadership that attends to both relationship maintenance and tasks goals are critical to the building and sustainment of effective CBPR partnerships to achieve health equity [31–35].

### Limitations

The data presented here reflect self-reported responses belonging to a small and purposive sample of active partnership members on the WB-HBV Project Task Force. Results may have been subjected to respondent and recall bias and are not generalizable. Nonetheless, they do provide recommendations to strengthen and structure similar ACG partnerships. Moreover, results can be compared to other partnerships that used this same survey [8]. However, the mixed-method study captured additional information that may have been missed quantitatively.

## Conclusions

This mixed-methods process evaluation on an ACG partnership offers critically important insights into developing strategies to enhance partnership functioning and increase the ability of this and future ACG partnerships to improve community health outcomes. The current process evaluation through a quantitative lens showed a general agreement among the three organizations across a variety of the measures adopted from Israel and colleagues, and the qualitative method allowed members to voice detailed comments regarding meeting the ultimate goal of eliminating HBV disparities in the WBMA. The results of the current process evaluation reveal strengths and weaknesses that may help to strengthen other ACG partnerships in the future.

## Data Availability

The datasets generated and/or analyzed during the current study are not publicly available due to confidentiality of participants but are available from the corresponding author on reasonable request.

## Abbreviations

WB-HBV: Washington-Baltimore Metropolitan Area Hepatitis B Virus
ACG: Academic-Community-Government
HBV: Hepatitis B Virus
HCV: Hepatitis C Virus
DC: District of Columbia
CBPR: Community-based Participatory Research
HCC: Hepatocellular Carcinoma
CDC: Centers for Disease Control and Prevention
REDCap: Research Electronic Data Capture
SPSS: Statistical Package for the Social Sciences
IRB: Institutional Review Board
COVID-19: Coronavirus 2019
EMR: Electronic Medical Record
NWP: Neighborhoods Working in Partnership
LGBT: Lesbian, Gay, Bisexual, and Transgender
PWID: People who Inject Drugs
